# First clinical cases of leishmaniosis in meerkats (*Suricata suricatta*) housed in wildlife parks in Madrid, Spain

**DOI:** 10.1186/s13071-024-06647-1

**Published:** 2025-01-28

**Authors:** Pablo Moraleda-Berral, Rosa Gálvez, Eva Martínez-Nevado, Lino Pérez de Quadros, Juncal García, Manuel de la Riva-Fraga, Juan Pedro Barrera, Efrén Estévez-Sánchez, Lourdes Cano, Rocío Checa, María Ángeles Jiménez-Martínez, Ana Montoya, Guadalupe Miró

**Affiliations:** 1 Departamento de Sanidad Animal, Facultad de Veterinaria, Universidad Complutense de Madrid, Avda. Puerta de Hierro S/N, 28040 Madrid, Spain; 2 Departmento de Didácticas Específicas, Facultad de Formación de Profesorado y Educación, Universidad Autónoma de Madrid, C. Francisco Tomás y Valiente 3, 28049 Madrid, Spain; 3 Zoo de Madrid, Casa de Campo S/N, 28011 Madrid, Spain; 4 Faunia, Avenida Comunidades 28, 28032 Madrid, Spain; 5 Departamento de Microbiología y Parasitología, Facultad de Farmacia, Universidad Complutense de Madrid, Pl. Ramón y Cajal S/N, 28040 Madrid, Spain

**Keywords:** Wild animals, Zoological parks, *Leishmania infantum*, Leishmaniosis, Meerkats, *Suricata suricatta*, Madrid, Sand flies

## Abstract

**Background:**

In recent years, cases of leishmaniosis have been described in animals housed in captivity in zoos in Spain [Bennett’s wallaby (*Macropus rufogriseus rufogriseus*), orangutan (*Pongo pygmaeus pygameus*), and European otter (*Lutra lutra*)]. Some of these zoological parks are in endemic areas for both human and animal leishmaniosis, thus it should be very important to include this zoonosis in the differential diagnosis.

**Methods:**

The study was carried out in two zoological parks in Madrid, Madrid Zoo and Faunia, and analyzed seven meerkats. Serological tests [rK-39 and enzyme-linked immunosorbent assay (ELISA)] and molecular tests [nested polymerase chain reaction (PCR) and real-time PCR] were performed to detect *Leishmania* DNA. Additionally, an entomological study was carried out in both zoological parks, with molecular tests performed on female *Phlebotomus perniciosus* sand flies to determine their blood meal source and detect *Leishmania* DNA.

**Results:**

Two meerkats were positive for *L. infantum*. A 9-year-old male from the Madrid Zoo died suddenly, showing pale mucous membranes and bilateral noninflammatory alopecia and hyperpigmentation in the lateral area of the eyes. Positive results were obtained in serology, nested PCR, and real-time PCR (blood, conjunctival and oral swabs, hair, spleen, lymph node, liver, kidney, and skin), as well as numerous amastigotes in the liver and kidney tissue samples. The other meerkat, a 12-year-old male from Faunia that is still alive, presented an alopecic lesion at the base of the tail. Positive results were obtained by nested and real-time PCR from different tissues such as blood, hair, oral, and conjunctival swabs. It was treated with oral allopurinol (25 mg/kg) and miltefosine (2 mg/kg), but the molecular diagnosis remained positive after 8 months, regarding it as a mild stage of the disease. The rest of the tested meerkats were negative. The presence of *P. perniciosus* phlebotomine sand flies was also detected in both zoos. Although no *L. infantum* DNA was detected in any of sand flies analyzed, it was determined that their food sources were rabbits and humans.

**Conclusions:**

To our knowledge, this study describes, for the first time, the detection and infection by *L. infantum* in meerkats (*Suricata suricatta*).

**Graphical Abstract:**

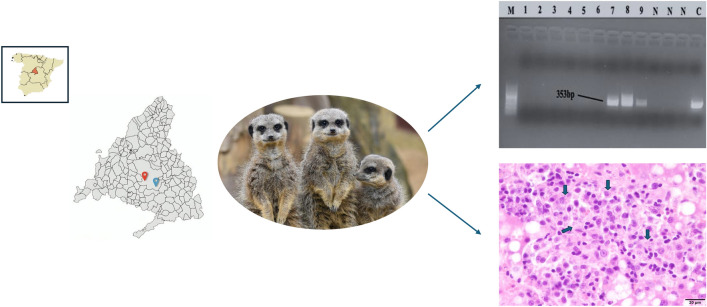

**Supplementary information:**

The online version contains supplementary material available at 10.1186/s13071-024-06647-1.

## Background

Leishmaniosis is a zoonotic vector-borne disease caused by the protozoan *Leishmania* spp., endemic in the Iberian Peninsula, which affects many mammals, including humans. In Spain, the dog (*Canis familiaris*) is considered the main peridomestic reservoir of the infection, which is transmitted through the bite of female phlebotomine sand flies [1, 2]. In addition to dogs and humans, *L. infantum* infection has been documented in other domestic and wild mammals in Spain [3], such as carnivores: brown bear (*Ursus arctos*) [4], cat (*Felis catus*) [5], ferret (*Mustela putorius furo*) [6], genet (*Genetta genetta*) [5], Iberian lynx (*Lynx pardinus*) [7], Iberian wolf (*Canis lupus signatus*) [5, 8], red fox (*Vulpes vulpes*) [9], mongoose (*Herpestes ichneumon*) [7, 10], bats (*Pipistrellus pipistrellus*) [11], European hedgehogs (*Erinaceus europaeus*) [3], Eurasian red squirrel (*Sciurus vulgaris*) [3], lagomorphs [5], and rodents [11]. Likewise, the infection has been reported in wild animals housed in captivity, such as Bennet’s wallabies (*Macropus rufogriseus rufogriseus*) in Faunia [12] and orangutans (*Pongo pygmaeus pygameus*) in the Madrid Zoo and Primates Rescue and Rehabilitation Center (Rainfer) [13]. Since then, more zoo veterinarians have included leishmaniosis as a differential diagnosis in animals living in endemic areas or coming from these areas, as has happened in the study of this disease in tigers (*Panthera tigris*) in a zoo in Italy [14] and with the first case of leishmaniosis in a European otter (*Lutra lutra*) in a zoo in Murcia [15]. Additionally, there have been reports of the first cases of leishmaniosis in two Patagonian maras (*Dolichotis patagonum*) in the Madrid Zoo [16].

The search for new reservoirs and the conduct of several studies of leishmaniosis in wild animals in Europe was triggered by the fact that in 2010, the largest outbreak of human leishmaniosis in Europe occurred in southwest Madrid, with rabbits and hares being the main reservoirs of leishmaniosis, rather than dogs [17, 18]. Therefore, xenodiagnostic and xenomonitoring studies have been carried out to search for new reservoirs [17] and better understand the sylvatic cycle. Two studies investigating the presence of sand flies have been conducted in wildlife parks in Spain, one at the zoo in Murcia [19] and another in Faunia [20], identifying *L. infantum* DNA in female sand flies in Faunia.

The aim of this study was to describe the infection by *L. infantum* in meerkats (*Suricata suricatta*) and to describe the first clinical cases of leishmaniosis in meerkats.

## Methods

### Study area

The study was carried out from December 2020 to January 2023 in two wildlife parks in the Madrid Autonomous Community, Madrid Zoo and Faunia (Additional file 1: Fig. S1). The Madrid Zoo (https://www.zoomadrid.com/) is in a wooded area in the southwest of the city of Madrid (40^o^ 25′ 22.2″ N, 3^o^ 45′ 32.0″ W). This zoological park houses more than 4000 animals of over 350 species, many of which are endangered.

Faunia (https://www.faunia.es/) is in the east of the Madrid Autonomous Community (40^o^ 39′ 38″ N, 3^o^ 61′ 24″ W) in a region that is drier and more arid compared with the Madrid Zoo. This zoological park has four areas recreating distinct natural ecosystems (jungle, temperate forest, African forest, and Antarctic) with lakes and ponds, housing more than 500 animal species, many of them with great conservation value.

### Study design

#### Host surveillance (active and passive)

In the present study, passive surveillance of *L. infantum* infection was conducted on meerkats housed in both zoological facilities, which were captured during routine procedures (e.g., deworming). Additionally, active surveillance was performed on animals exhibiting clinical signs compatible with *L. infantum* infection (e.g., cutaneous lesions) or on those that had died suddenly.

### Study animals

In total, seven meerkats, five from the Madrid Zoo and two from Faunia, were analyzed. Of these, four were males and three were females. All of them were born in the Madrid Zoo, except for one meerkat from a school farm, which was born in Fuenlabrada, in the south of the Madrid Autonomous Community. During the study period, samples were taken from five animals after they had died (cases: 1–5) and two are still alive (cases: 6–7). Cases 1–5 belonged to the Madrid Zoo, while cases 6–7 belonged to Faunia.

#### Sample collection

Samples collected from live animals were taken during the veterinary procedures. Before the physical examination and sampling, the animals were sedated with a combination of ketamine (10 mg/kg) and midazolam (0.5 mg/kg) for those belonging to Faunia, while for those from the Madrid Zoo, a combination of ketamine (5 mg/kg) and medetomidine (0.1 mg/kg) was used. If it was necessary, they were intubated and maintained with isofluorane. Peripheral blood (2 ml) was collected from the cranial cava vein into two tubes: (a) EDTA (1 ml) for *Leishmania* DNA detection by PCR and (b) a tube without additives (1 ml) for serological tests to detect anti-*L. infantum* antibodies and anti-*Toxoplasma gondii* antibodies. In addition, oral, conjunctival, ear, and genital mucosa swabs and hair were collected to detect *Leishmania* DNA by PCR.

In the case of the death of an animal, samples of spleen, liver, lymph node, kidney, and skin were collected post mortem after necropsy, and peripheral blood, serum, swabs, and hair were also collected from some of them. All samples were stored at −20 °C until processing for later DNA extraction and molecular diagnosis. In addition, the main tissue samples from cases 1, 3, and 4 were stored in 10% buffered formalin for histopathology, following standard laboratory procedures.

### Serological tests

For the detection of anti-*L. infantum* antibodies, a commercial immunochromatography technique based on the detection of the recombinant rK39 antigen (rK39 RDT Kalazar Detect, InBios International, Seattle, USA) was used. Additionally, an enzyme immunoassay (ELISA) was performed for the qualitative determination of antibodies against *Leishmania* (VetLine *Leishmania* ELISA, NovaTec Immunodiagnostica GmbH, Dietzenbach, Germany) in serum samples from the meerkats, using a cut-off > 11 NovaTecs Units(NTU) to define seropositivity.

For the qualitative determination of specific immunoglobulin G (IgG) anti-*T. gondii* antibodies in serum samples from the meerkats, the NovaTec VetLine Toxoplasma ELISA was used (VetLine *Toxoplasma* ELISA, NovaTec Immunodiagnostica GmbH, Dietzenbach, Germany), using a cut-off > 55 IU/ml to consider seropositivity.

### Molecular diagnosis

For the detection of *Leishmania* DNA in biological samples obtained from the same animals, nested PCR and real-time PCR were used [21, 22].

Previously, DNA extraction from the biological samples was performed using the QIAamp DNA Mini Kit (QIAGEN, Hilden, Germany), following the protocols defined by the manufacturer. The obtained DNA was eluted in 200 μl of distilled water for blood and tissue samples and 150 μl for swabs. DNA samples were stored at −20 °C until use.

For the detection of *Leishmania*, a 20 μl aliquot of eluted DNA was used for each nested PCR and a 10 μl aliquot of eluted DNA for each real-time PCR. The nested PCR protocol was carried out following the protocol described by Cruz et al. [21] that amplifies a variable region of the SSUrRNA gene, with some modifications.

Real-time PCR protocol was performed, following the one described by Chicharro et al. [22], which detects conserved regions of the 18S rRNA gene and allows for the determination of the parasitic load in the sample analyzed. Samples showing a threshold cycle (CT) value of 37 or lower were considered positive in the study [22].

Nested PCR amplification products that were visualized as a single intense band in the agarose gels and with the expected size according to the amplified region [353 base pairs (bp)], were included in tubes for subsequent shipment to the sequencing service of the Genomics Unit at the Universidad Complutense de Madrid, where they underwent purification and subsequent sequencing. DNA sequencing was carried out bidirectionally (sense and antisense sequences) using the same primers that were used for the amplification of the different regions of interest previously exposed. The reading of each sequence was carried out in an ABI Prism 3730 system automatic sequencer (Applied Biosystems). The sequences obtained were analyzed and edited using the MEGA 11 software [23]. Edited DNA sequences were compared with those available in GenBank using BLAST (Basic Local Alignment Search Tool).

### Necropsy and histopathological exam

A systematic necropsy was performed, and well-preserved tissue samples were sent to the Zoo and Wildlife Pathology Service of the Complutense University Veterinary Teaching Hospital. Samples (spleen, liver, kidney, heart, small and large intestine, penis, pancreas, urinary bladder, trachea, and lungs) were processed for histopathology, following routine laboratory procedures, and examined by a trained pathologist in cases 1, 3, and 4.

### Vector surveillance

#### Entomological survey

During May to October, in the years 2019–2020, sticky traps (A5-size paper coated with castor oil) were placed in the Madrid Zoo and during the same months, but from 2020–2021, sticky traps were placed in Faunia. All traps were placed in the morning in the meerkat facilities in strategic locations, preventing animals from accessing them (as in wall holes), and were collected on days 3 or 4 after laying. Collected sand flies were kept in 70% ethanol until processing. Females were cleared in Mark André medium and mounted on glass slides in Hoyer medium [24]. Species classification was performed according to identification keys [25]. In addition, the abdomen was stored in 70% ethanol until molecular diagnosis.

### Molecular diagnosis

#### DNA extraction

For the detection of *Leishmania* DNA, DNA was extracted from the abdomen of captured *P. perniciosus* females using QIAamp DNA Mini Kit (QIAGEN, Hilden, Germany).

### Molecular tests

For the detection of *Leishmania* DNA from the pool of female sand flies captured at the Madrid Zoo and Faunia, real-time PCR was used following the protocol described by Chicharro et al. [22].

For the detection of mammalian blood DNA, direct PCR was used following the protocol of Abbasi et al. [26], with some modifications. The analysis of blood meal preferences was done by amplification of a 359 bp fragment of cytochrome b gene, followed by sequence analysis using the MEGA 11 software [23] and BLAST for identification through homology search in the GenBank database.

## Results

Of the seven meerkats analyzed, *Leishmania* was detected by serology and/or PCR in two individuals (cases 1 and 7). One of these meerkats (case 1) died, while the other (case 7) remains alive. Case 1 showed cutaneous lesions compatible with leishmaniosis (as described above) and died suddenly. Case 2 was found mummified in one of the burrows, with the date and cause of death unknown. In case 3, no *L. infantum* DNA was detected by nested PCR and real-time PCR, and no compatible lesions were observed. Cases 4 and 5 were analyzed while alive (cases 4a and 5a) and again after their deaths (cases 4b and 5b). Case 6 remains alive and was analyzed without showing any lesions compatible with leishmaniosis. Case 7 is currently alive and has a cutaneous lesion on the tail compatible with leishmaniosis. All of these cases are described below and are summarized in Tables 1 and 2, where the results of the analyzed samples, both PCR and serology, are indicated.

**Table 1:** Samples obtained from live and dead meerkats and serological results for *Leishmania infantum* and *Toxoplasma gondii*

Case	Animal ID	Sex	Age (*years)*	Zoo park	Origin (*birth)*	Status	Serology results		
							*RK-39*	ELISA *Leishmania infantum*	ELISA *Toxoplasma gondii* (IgG)
1	SUR-58	M	9	Madrid Zoo	Madrid Zoo	Dead	**Pos**	**Pos**	Neg
2	SUR-46	F	10	Madrid Zoo	Madrid Zoo	Dead	Nt	Nt	Nt
3	SUR-43	M	10	Madrid Zoo	Madrid Zoo	Dead	Neg	Neg	Neg
4a	SUR-65	F	7	Madrid Zoo	Madrid Zoo	Alive	Neg	Neg	Neg
4b	SUR-65	F	7	Madrid Zoo	Madrid Zoo	Dead	Nt	Nt	Nt
5a	SUR-42	M	11	Madrid Zoo	Madrid Zoo	Alive	Neg	Neg	Neg
5b	SUR-42	M	11	Madrid Zoo	Madrid Zoo	Dead	Nt	Nt	Nt
6	033/20	F	5	Faunia	Fuenlabrada	Alive	Neg	Neg	Neg
7a	008/19	M	12	Faunia	Madrid Zoo	Alive	Nt	Nt	Nt
7b	008/19	M	12	Faunia	Madrid Zoo	Alive	Neg	Neg	**Pos**

F, female; M, male; Neg, negative; **Pos**, positive; Nt, not tested

**Table 2:** Samples obtained from live and dead meerkats and molecular results for *Leishmania infantum*

Case	Animal ID	Zoo park	Status	Nested PCR results											*q*PCR results										
				PB	OS	CS	ES	GS	H	LV	SP	KD	SK	LN	PB	OS	CS	ES	GS	H	LV	SP	KD	SK	LN
1	SUR-58	Madrid Zoo	Dead	**Pos**	**Pos**	**Pos**	Neg	Nt	**Pos**	**Pos**	**Pos**	**Pos**	**Pos**	**Pos**	**Pos**	Nt	Nt	Nt	Nt	**Pos**	Nt	**Pos**	Nt	Nt	Nt
2	SUR-46	Madrid Zoo	Dead	Nt	Nt	Nt	Nt	Nt	Neg	Nt	Nt	Nt	Neg	Nt	Nt	Nt	Nt	Nt	Nt	Nt	Nt	Nt	Nt	Nt	Nt
3	SUR-43	Madrid Zoo	Dead	Neg	Neg	Neg	Neg	Neg	Neg	Neg	Neg	Neg	Neg	Neg	Neg	Neg	Neg	Neg	Neg	Neg	Neg	Neg	Neg	Neg	Neg
4a	SUR-65	Madrid Zoo	Alive	Nt	Neg	Neg	Neg	Neg	Neg	Nt	Nt	Nt	Nt	Nt	Nt	Nt	Nt	Nt	Nt	Nt	Nt	Nt	Nt	Nt	Nt
4b	SUR-65	Madrid Zoo	Dead	Nt	Neg	Neg	Neg	Nt	Neg	Neg	Neg	Neg	Neg	Neg	Nt	Nt	Nt	Nt	Nt	Nt	Nt	Nt	Nt	Nt	Nt
5a	SUR-42	Madrid Zoo	Alive	Neg	Neg	Neg	Neg	Neg	Neg	Nt	Nt	Nt	Nt	Nt	Neg	Nt	Nt	Nt	Nt	Nt	Nt	Nt	Nt	Nt	Nt
5b	SUR-42	Madrid Zoo	Dead	Nt	Nt	Nt	Nt	Nt	Neg	Nt	Nt	Neg	Nt	Nt	Nt	Nt	Nt	Nt	Nt	Nt	Nt	Nt	Nt	Nt	Nt
6	033/20	Faunia	Alive	Neg	Nt	Nt	Nt	Nt	Nt	Nt	Nt	Nt	Nt	Nt	Neg	Nt	Nt	Nt	Nt	Nt	Nt	Nt	Nt	Nt	Nt
7a	008/19	Faunia	Alive	**Pos**	**Pos**	**Pos**	Nt	Neg	Nt	Nt	Nt	Nt	Nt	Nt	**Pos**	**Pos**	**Pos**	Nt	Neg	Nt	Nt	Nt	Nt	Nt	Nt
7b	008/19	Faunia	Alive	**Pos**	**Pos**	**Pos**	Nt	Nt	**Pos**	Nt	Nt	Nt	Nt	Nt	**Pos**	**Pos**	**Pos**	Nt	Nt	Nt	Nt	Nt	Nt	Nt	Nt

F, female; M, male; PB, peripheral blood; OS, oral swab; CS, conjunctival swab; ES, ear swab; GS, genital swab; H, hair; LV, liver; SP, spleen; KD, kidney; SK, skin; LN, lymph node; Neg, negative; **Pos**, positive; Nt, not tested

### Case 1

**Fig. 1:**
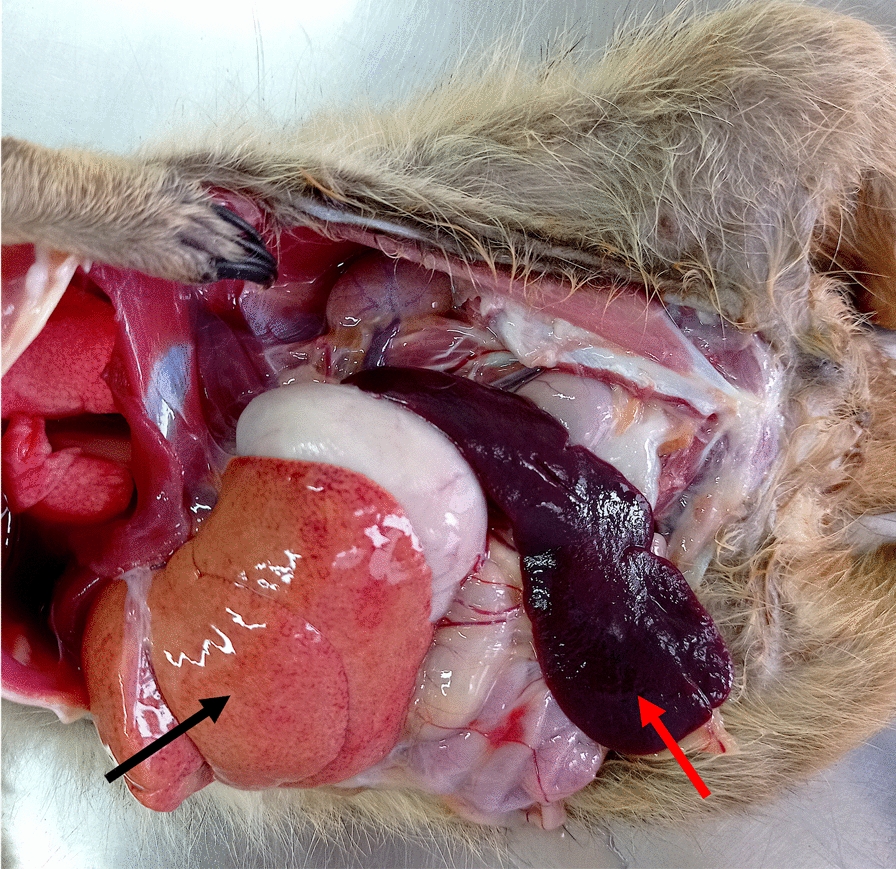
Necropsy results of the meerkat from case 1 showing splenomegaly (red arrow) and hepatic discoloration (black arrow)

**Fig. 2:**
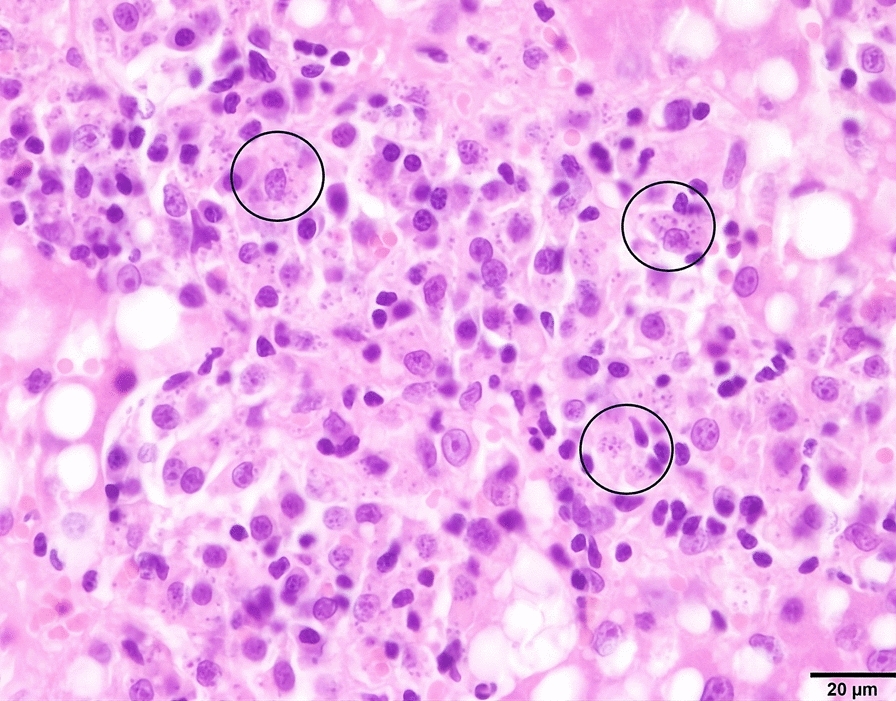
Histopathology results of the meerkat from case 1. Liver, hematoxilin&eosin. Multifocal to coalescing aggregates of macrophages, admixed lymphocytes and neutrophils, displacing hepatocytes. Many macrophages contain numerous 2 µm amastigotes with perpendicular kinetoplasts (circles)

A 9-year-old male meerkat, born in the Madrid Zoo and having always lived in this park, died suddenly on 23 December 2020, 1 day after presenting apathy, inactivity, and dyspnea. The animal presented paleness of the mucous membranes and bilateral alopecia with hyperpigmented skin in the lateral area of the eyes. At necropsy, the animal had pale mucous membranes, splenomegaly, a diffusely pale orange, mildly enlarged liver, and lymphadenomegaly (Fig. 1). A rapid immunochromatographic test (ICT) was performed on the serum on the basis of the detection of the rK39 recombinant antigen, yielding positive results. Likewise, the amount of anti-*Leishmania* antibodies was quantified using the ELISA technique, obtaining a positive result of 12.42 NTU. Nested PCR was performed on all of the DNA samples, resulting in positive results for DNA from blood, oral and conjunctival swabs, liver, spleen, kidney, lymph node, and skin biopsies (Additional file 1: Fig. S2). Additionally, real-time PCR was performed on DNA from blood, hair, and spleen samples, again yielding positive results (TC values of 20.79, 32.27, and 16.04, respectively). The histopathological examination revealed severe, multifocal to coalescing granulomatous hepatitis, splenitis, nephritis, and pneumonia, with myriads of intralesional (2–4 micron) uninucleate protozoa with a perpendicular kinetoplast, consistent with amastigotes (Fig. 2). The remaining organs were within normal limits. Skin and lymph nodes were not collected for histopathology in this case. Death was determined to be a direct consequence of the severe infection. Furthermore, ELISA against *T. gondii* was performed as a differential diagnosis, yielding a negative result.

### Case 2

In June 2021, the mummified body of a meerkat was discovered at the Madrid Zoo, with the cause and date of death remaining unknown. Only hair and skin samples could be obtained, both of which tested negative for *Leishmania* by nested PCR and real-time PCR.

### Case 3

In October 2021, a 10-year-old male meerkat died, having lived its entire life at the Madrid Zoo. Both the rK39 and ELISA tests for *Leishmania* spp. were negative. All samples tested were negative by nested PCR and real-time PCR. Furthermore, histopathological examination revealed a hepatocellular carcinoma causing chronic hemorrhage, which weakened the animal and ultimately led to its death. No forms compatible with *Leishmania* were observed. Additionally, an ELISA test for *T. gondii* was performed, with a negative result.

### Cases 4 to 5

In January and July of 2022, a veterinary intervention was performed on a 7-year-old female and an 11-year-old male meerkat, respectively, both born at the Madrid Zoo. Swabs and hair samples were collected from both animals, and peripheral blood and serum samples were collected from the male. All samples tested negative for *L. infantum*. In March and September of the same year, both meerkats died, and all samples collected post-mortem were also negative for *L. infantum*. Furthermore, histopathological examination for case 4 revealed that the cause of death in the female meerkat was chronic renal disease accompanied by severe cardiac failure. For case 5, the cause of death could not be determined, as only skin and kidney samples were available. No forms compatible with *Leishmania* were observed in either case. Additionally, an ELISA test for *T. gondii* was performed, with a negative result.

### Case 6

In September 2022, a routine veterinary procedure was performed on a 5-year-old female meerkat living in Faunia, originally from a school farm in Fuenlabrada (southwest Madrid). The anesthesia protocol employed included ketamine (Ketamidor, Karizoo, Spain) at a dosage of 10 mg/kg and midazolam (Midazolam, Accord Healthcare, Spain) at 0.5 mg/kg as sedatives, with isofluorane (IsoFlo, Ecuphar, Spain) used as the inducer [27]. The meerkat was treated for fleas with nitenpyram (Capstar, Elanco GmbH, Germany) upon arrival at Faunia, as a preventive measure, and a general coprological analysis was performed, yielding negative results. Peripheral blood and serum samples were collected, both of which tested negative for *L. infantum*. Additionally, an ELISA test for *T. gondii* was performed, obtaining a negative result.

### Case 7a and 7b

In January 2023, a 12-year-old male meerkat from Faunia underwent a veterinary procedure of a radiographic examination of the left hind limb. It was born and spent time at the Madrid Zoo until it was 7 years old. The meerkat was anesthetized with ketamine (Ketamidor, Karizoo, Spain) (10 mg/kg) and midazolam (Midazolam, Accord Healthcare, Spain) (0.5 mg/kg) as sedatives and isofluorane (IsoFlo, Ecuphar, Spain) as the inducer, owing to a lameness in the left hind limb. It was also observed that the subject had an alopecic lesion at the base of the tail, which had been dragging since the meerkat was at the Madrid Zoo. The lesion identified in March 2019 did not exhibit signs of infection, and all fungal cultures performed return negative results. Peripheral blood samples and oral, conjunctival, and genital mucosa swabs were taken, being positive for *L. infantum* in DNA samples from blood, oral and conjunctival swabs using nested PCR and real-time PCR (CT values of 29.2, 30.47, and 31.11, respectively). The meerkat was treated against *L. infantum* in March 2023 with miltefosine (Milteforan, Virbac, Carros, France) at an empirical dose of 2 mg/kg orally once a day for 28 days, and since June 2023, allopurinol was administered once a day for a month (Zyloric, Faes Farma S.A., Bizkaia, Spain) at 25 mg/kg, being an empirical dose.

In September 2023, the animal was recaptured for disease control, again taking samples of peripheral blood, serum, and oral and conjunctival mucosa swabs, as well as hair from the injured area of the tail. There was no observed improvement in the lesion at the base of the tail. Nested PCR returned positive results for DNA samples from peripheral blood, hair, and oral and conjunctival mucosa swabs. The real-time PCR detected positive results in the same DNA samples, except for the hair samples, showing TC values of 29.18, 27.73, and 27.19, respectively. Both the rK39 and ELISA serological tests for detecting antibodies against *Leishmania* spp. were negative. Furthermore, an ELISA against *T. gondii* was performed as a differential diagnosis, obtaining a positive result in IgG (133.5 IU/ml). Hematology was also performed, showing mild anemia, leukopenia, neutropenia, and lymphopenia.

### Sequencing results

All sequences obtained from PCR-positive DNA samples were 100% identical to *L. infantum*. Consensus sequences were submitted to the GenBank database under the accession numbers: PQ114156 and PQ114157. This causative agent was also identified in recently reported cases of the infection in wild micromammals in northwest Spain [28].

### Entomological survey

During sampling at the Madrid Zoo between May and October in 2019 and 2020, 170 sticky traps were set and 164 were collected. A total of 570 phlebotomine sand flies were captured, 261 females (45.8%) and 309 males (54.2%). Regarding the species of phlebotomine sand flies captured and identified, *P. perniciosus* (46 specimens; 8.1%) and *Sergentomyia minuta* (524 specimens; 91.9%) were collected, 8 females (1.4%) and 38 males (6.7%) of the species *P. perniciosus* and 253 females (44.4%) and 271 males (47.5%) of the species*S. minuta*.

During the study, a total of 122 sticky traps were placed in Faunia and 113 traps were collected between May and October in 2020 and 2021 (Additional file 1: Fig. S3). A total of 128 phlebotomine sand flies were captured and identified as *P. perniciosus* (11 specimens; 8.6%), and *S. minuta* (117 specimens; 91.4%). Regarding sex distribution, of the total number of *P. perniciosus* specimens, 3 were female (2.3%) and 8 male (6.3%), and of the species *S. minuta*, 28 were female (21.9%) and 89 male (69.5%).

Owing to adverse weather conditions, including heavy rain and strong winds, not all sticky traps could be retrieved from both zoos during the sampling days.

All tested *P. perniciosus* females from the Madrid Zoo and Faunia were negative for *L. infantum*, as determined by real-time PCR.

The cytochrome b amplification for identifying feeding sources resulted positive in the blood meal of seven fed females of the species *P. perniciosus*. Sequencing results revealed that two out of seven females had fed on rabbit blood (*Oryctolagus cuniculus*), while five had fed on human blood (*Homo sapiens sapiens*).

## Discussion

To the best of our knowledge, this report describes, for the first time, cases of leishmaniosis in meerkats. There are references in the *Herpestidae* family in Portugal [29] and Spain [7, 10], where leishmaniosis is endemic, although they did not present any lesion compatible with the disease.

Despite the confirmation of clinical leishmaniosis in the meerkats at the Madrid Zoo and Faunia, the role of this species in the transmission of the disease is still unknown. Although xenomonitoring studies have been carried out in peri-urban wildlife parks in southeastern Spain [19, 30] and southern Italy [14] to determine the food source of sand flies, further xenodiagnostic studies are necessary [29].

The rapid onset of clinical signs in case 1 did not allow for the monitoring of the disease and determining treatment, as has been done in other cases of leishmaniosis in zoo animals [13, 15]. Similarly, cases of toxoplasmosis in meerkats have exhibited a sudden disease progression, resulting in fatalities within a few days and leaving little opportunity for intervention [31, 32]. This highlights their susceptibility to diseases caused by intracellular protozoa. Further studies are warranted to investigate the immune response of meerkats to these pathogens to better understand their defensive capabilities.

The kinetoplasts observed within the protozoa in the histopathological analysis are not always easily identifiable in tissue sections and may be easily mistaken for other agents such as *T. gondii*. Therefore, considering our results, leishmaniosis should be included as a differential diagnosis when encountering intracellular protozoa in meerkats. Necropsy findings are common in other cases of visceral leishmaniosis in wild and captive-housed animals [33], as well as alopecia, which is particularly observed in dogs and wild canids [34].

In case 7, while serological tests were negative for *L. infantum* antibodies, molecular analyses detected DNA of the parasite in various samples. This discrepancy may be owing to the immune response of the meerkat, which appears competent despite ongoing infection, as occurs in dogs and cats [35, 36], suggesting it fits the criteria for a clinically infected stage I animal according to canine leishmaniosis guidelines [2]. Follow-up should be performed every 3–6 months, with serological testing deferred for at least 6 months [2]. The lack of response to leishmanicidal and leishmaniostatic treatments may be owing to limited data on their efficacy in meerkats, such as findings in other domestic species [37]. Concurrent diseases, as seen in dogs and cats, could also be a contributing factor [2, 35]. While infectious processes were ruled out during the study, noninfectious factors such as trauma or self-harm may still play a role, which is frequent in zoo animals [38]. Furthermore, the presence of IgG antibodies against *Toxoplasma* suggests prior exposure to the parasite, likely resulting from nearby rodent and feral cat populations, as well as potential food contamination with cat feces, consistent with findings in meerkats at the Johannesburg Zoo in South Africa [39].

Meerkats are carnivorous mammals belonging to the *Herpestidae* family, native to southern Africa. These social animals typically inhabit large, female-dominated groups and are commonly found in zoos, where they have access to both burrows and outdoor areas [40]. As diurnal diggers, meerkats create extensive underground galleries, which can increase their exposure to biting sand flies. These insects prefer dark, humid environments for resting, conditions [41] frequently found within meerkat burrows. Additionally, the presence of organic matter, sparse vegetation, and sandy soils within these galleries may attract a higher density of sand flies, which seek refuge from rain and wind owing to their limited flying ability. This behavior aligns with findings from studies that have examined sand fly populations in similar burrow environments [42, 43].

There were studies carried out at the Madrid Zoo and Faunia where the presence of two sand fly species (*P. perniciosus* and *S. minuta*) was detected [12, 13]. While female sand flies were found in meerkat facilities, no meerkat blood was detected in fed females, despite reports of blood from humans and rabbits. This may be owing to the preference of *P. perniciosus* for humans [30] and the common presence of field rabbits in both parks, as these vectors are known to favor lagomorphs [44]. Additionally, feeding preferences may vary on the basis of factors such as distance of traps from meerkat burrows and seasonal availability of preferred host [30].

The presence of fed females could mean a possible risk of local transmission of the parasite from these females to captive animals in these zoos, as seen in cases of leishmaniasis in Bennet’s wallabies [12] or Bengal tigers [14]. However, the absence of *Leishmania* spp. detection in female sand flies could be owing to the performance of the nested PCR technique, whereas real-time PCR seems to increase diagnostic sensitivity [45].

Despite southern Africa (Namibia, Botswana, and South Africa) being the natural habitat of meerkats, there is hardly any work on sand flies and the disease [46, 47], which may be because it has not been considered a risk owing to the low number of reported cases. The presence of parasite-transmitting sand flies has been recorded, as well as reservoirs such as rodents [46, 47]. It would be interesting to carry out work on wildlife, including meerkats, to determine the role they may have on the disease in their place of origin.

Future studies are essential to determine the blood preferences of sand flies and to understand their role in transmitting this zoonotic disease. It is crucial to investigate whether these animals may be affected by the disease, as observed in these cases, or whether they may act as reservoirs.

Although there is not a great conservation concern, according to the International Union for Conservation of Nature (IUCN) [48], this disease should be included in the differential diagnosis in zoos located in leishmaniasis endemic areas to preserve their conservation status, as was done in the cases of orangutans in Madrid [13]. Further work is needed to prevent *L. infantum* infection in this species through early diagnosis and the use of preventive measures in these environments as well as in the animals. Further entomological studies are needed to design effective control measures for insecticides applied to the environment. In addition, in wild animals in zoos, it is also necessary to control stress and any disease that may compromise the immune system and lead to clinical leishmaniasis in previously infected healthy animals [49].

The rapidity and lethality of case 1, the treatment unresponsiveness in case 7, and the appearance of deceased specimens in inaccessible galleries as seen in case 2 underscore the unknown causes of death. These animals are difficult to handle owing to their tendency to hide and their aggressive behavior when disturbed. Consequently, further studies on the disease in meerkats are crucial, as leishmaniosis may lead to significant specimen loss in zoos and a problem with their conservation, similar to the cases of Bennet’s wallabies (*Macropus rufogriseus rufogriseus*) at the Faunia zoo in Madrid [12], Timber wolves (*Canis lupus occidentalis*) at a zoo in Almeria, and the bush dogs (*Speothos venaticus*) in the Belo Horizonte zoo in Brazil [34], among other cases [13, 50].

Owing to these two cases (1 and 7) and others reported in different species housed in zoos [14, 15, 51], along with the presence of sand flies [12, 13], which thrive under ideal conditions in these parks [19], it is essential to establish control and prevention measures against the parasite, through a combination of strategies.

Vector control measures include the use of repellent plants such as lavender or citronella, although their effectiveness may be limited at present [41, 52]. Environmental strategies aimed at reducing organic matter are also recommended [41]. Likewise, fipronil baits administered to *Rhombomys opimus* rodents, known reservoirs of *L. major* in Central Asia, have significantly decreased the abundance of gravid females [53]. Furthermore, cypermethrin was found to be effective in controlling *Lutzomyia longipalpis* sand flies when sprayed on the walls of human residences in Brazil [54].

The preventive measures used in dogs against sandfly biting are the use of repellent products containing pyrethroids, such as collars or spot-on pipettes [18, 41]. However, none of the insecticide treatments available on the market against sand flies have 100% effective repellent activity [55]. In meerkats, these methods are complicated to use because there are no records of the use of synthetic pyrethroids and they could cause toxicity, as seen in ferrets [56]. Imidacloprid has been safely used topically in combination with moxidectin (Advocate^®^, Bayer Animal Health) in a group of meerkats in a zoo in Zurich, Switzerland [40], and it has been reported that imidacloprid may be an effective insecticide against sand flies [41]. The use of flumethrin, a synthetic pyrethroid, as a phlebotomine repellent agent has been used safely in cats [57]. However, studies on the safety and effectiveness of this repellent drug in meerkats are needed.

## Conclusions

According to the consulted bibliography, these are the first diagnosed records of clinical leishmaniosis cases in meerkats in a zoo. Owing to the presence of sand flies in their area, as well as the clinical signs presented, we must consider meerkats as susceptible animals to becoming infected and developing leishmaniasis in endemic areas; however, their role as secondary reservoirs is still not demonstrated.

## Supplementary information

Additional file 1: Figure S1. Map showing the location of the Zoo Aquarium of Madrid and Faunia, in the Community of Madrid, Spain. Figure S2. Positive nested PCR to *L. infantum* from the meerkat of case 1. Lane 1 to 6 are negative results, lane 7 to 9 are spleen, kidney, and liver positive samples. Lane 10 to 12 are negative controls. Lane 13 is positive control. M = molecular size marker. Figure S3. Placement of sticky traps in Faunia’s meerkat facility.

## Data Availability

No datasets were generated or analyzed during the current study.

## Abbreviations

BLAST: Basic local alignment search tool
TC: Threshold cycle
EDTA: Ethylenediaminetetraacetic acid
ELISA: Enzyme-linked immunosorbent assay
ICT: Immunochromatographic test
IU: International units
IUCN: International Union for Conservation of Nature
NTU: NovaTecs units
PCR: Polymerase chain reaction
