# Supplementary figures and images for: First clinical cases of leishmaniosis in meerkats (Suricata suricatta) housed in wildlife parks in Madrid, Spain
Source: Parasit Vectors. 2025 Jan 28;18:31. doi: 10.1186/s13071-024-06647-1 (PMC11773741; doi:10.1186/s13071-024-06647-1)

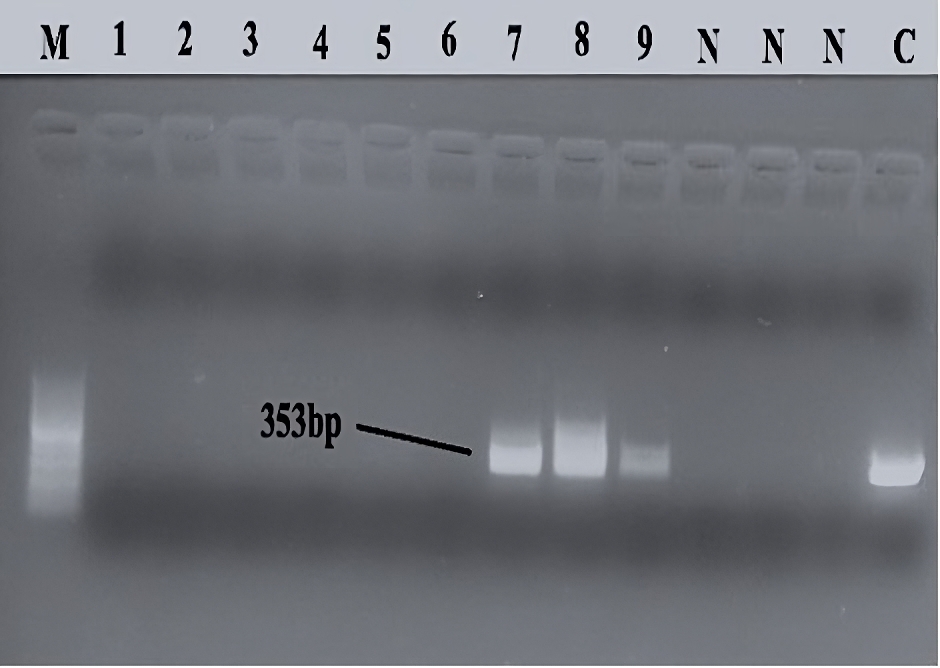

Supplement: Supplementary file 1 — Additional file 1: Figure S1. Map showing the location of the Zoo Aquarium of Madrid and Faunia, in the Community of Madrid, Spain. Figure S2. Positive nested PCR to L. infantum from the meerkat of case 1. Lane 1 to 6 are negative results, lane 7 to 9 are spleen, kidney, and liver positive samples. Lane 10 to 12 are negative controls. Lane 13 is positive control. M = molecular size marker. Figure S3. Placement of sticky traps in Faunia’s meerkat facility. [file 13071_2024_6647_MOESM1_ESM.zip › Figure_S2 Moraleda et al 24.jpeg]

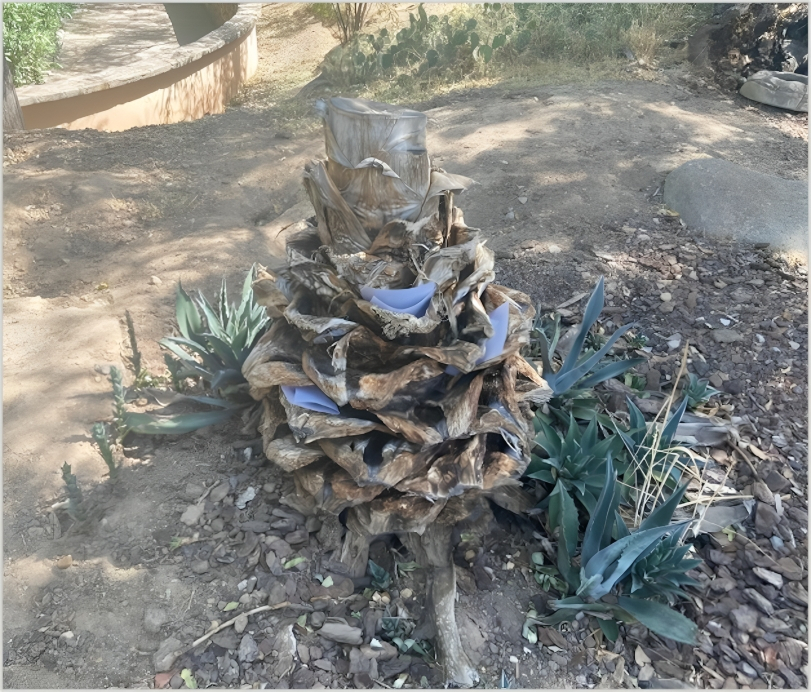

Supplement: Supplementary file 1 — Additional file 1: Figure S1. Map showing the location of the Zoo Aquarium of Madrid and Faunia, in the Community of Madrid, Spain. Figure S2. Positive nested PCR to L. infantum from the meerkat of case 1. Lane 1 to 6 are negative results, lane 7 to 9 are spleen, kidney, and liver positive samples. Lane 10 to 12 are negative controls. Lane 13 is positive control. M = molecular size marker. Figure S3. Placement of sticky traps in Faunia’s meerkat facility. [file 13071_2024_6647_MOESM1_ESM.zip › Figure_S3 Moraleda et al 24.jpg]

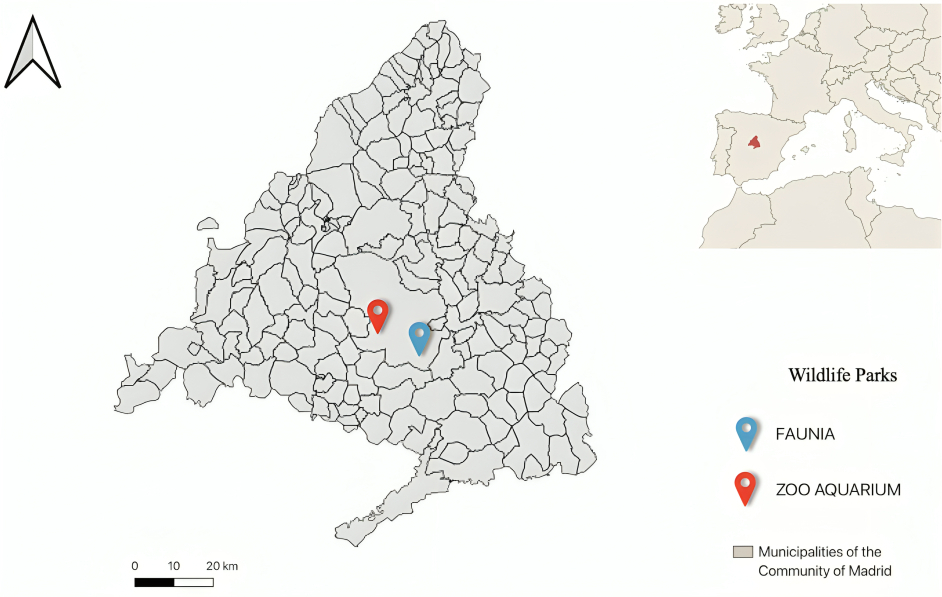

Supplement: Supplementary file 1 — Additional file 1: Figure S1. Map showing the location of the Zoo Aquarium of Madrid and Faunia, in the Community of Madrid, Spain. Figure S2. Positive nested PCR to L. infantum from the meerkat of case 1. Lane 1 to 6 are negative results, lane 7 to 9 are spleen, kidney, and liver positive samples. Lane 10 to 12 are negative controls. Lane 13 is positive control. M = molecular size marker. Figure S3. Placement of sticky traps in Faunia’s meerkat facility. [file 13071_2024_6647_MOESM1_ESM.zip › Figure_S1 Moraleda et al 24.jpg]
